# Pathological complete response to conversion therapy for lung adenocarcinoma with brain metastasis: a case report

**DOI:** 10.3389/fonc.2025.1625918

**Published:** 2025-10-14

**Authors:** Xingqiang Ran, Tao Luo, Jie Xiong, Maoyong Fu

**Affiliations:** Department of Thoracic Surgery, Affiliated Hospital of North Sichuan Medical College, Nanchong, China

**Keywords:** lung adenocarcinoma, brain metastasis, furmonertinib, conversion therapy, pathological complete response

## Abstract

Lung cancer is the leading cause of cancer mortality worldwide. Fortunately, the advent of precision medicine, which includes targeted therapy and immunotherapy, has significantly improved the survival rates of patients with locally advanced lung cancer. This article reports on a case of stage IVB (cT2bN1M1c1) non-small cell lung cancer (NSCLC) with brain metastases harboring compound mutations in epidermal growth factor receptor (EGFR) exon 21 Leu858Arg and mitogen-activated protein kinase 1 (MEK1) exon 3 lle112Thr and with a high program death ligand 1 (PD-L1) expression that successfully underwent radical lung cancer surgery following combined therapy. We report on a case of a 60-year-old man diagnosed preoperatively with stage IVB adenocarcinoma of the left upper lung (cT2bN1M1c1) who was diagnosed with multiple brain metastases. After multidisciplinary discussion, it was decided to administer targeted therapy with furmonertinib, chemotherapy with pemetrexed and lobaplatin, and immunotherapy with tislelizumab. Following 2 months of treatment, tumor assessment showed partial response (PR). After 11 months, assessment showed a PR of all lung lesions and complete response of the brain lesions, making the patient eligible for surgery. Finally, the patient underwent video-assisted thoracoscopic left upper lobectomy + mediastinal lymphadenectomy. Postoperative pathology confirmed complete response, and the patient continued adjuvant therapy with furmonertinib. For patients with metastatic advanced NSCLC, systemic treatment involving chemotherapy plus immunotherapy and targeted therapy is expected to become one of the options. Moreover, it is likely to achieve successful conversion surgery and further efficacy after combined therapy.

## Introduction

In approximately 40%–60% of Chinese patients with non-small cell lung cancer (NSCLC), mutations in the *EGFR* gene are present, enabling them to benefit from treatments involving epidermal growth factor receptor tyrosine kinase inhibitors (EGFR TKIs). Furmonertinib mesylate (AST2818) represents an innovative third-generation EGFR TKI with irreversible action. This medication has gained approval from the China National Medical Products Administration for use in individuals with locally advanced or metastatic NSCLC who have sensitive EGFR mutations along with the T790M resistance mutation (1). Due to its distinct trifluoroethoxypyridine configuration, furmonertinib exhibits an enhanced safety profile. Furthermore, both furmonertinib and its primary metabolite, AST5902, have demonstrated significant antitumor efficacy and excellent selectivity (2). Tislelizumab, an engineered immunoglobulin G4 (IgG4) monoclonal antibody targeting programmed cell death protein 1 (PD-1), has been meticulously developed to reduce attachment to FC gamma receptors (FcγR) on macrophages. The RATIONALE-307 trial assessed the safety and effectiveness of combining chemotherapy with tislelizumab as an initial treatment for patients with advanced NSCLC, revealing that this combination resulted in a median progression-free survival (PFS) exceeding 7.6 months. In addition, the frequency of adverse events (AEs) observed was comparable between the experimental group and the control group. This study supports the use of tislelizumab in conjunction with chemotherapy as the primary treatment for advanced squamous NSCLC as granted by the National Medical Products Administration (NMPA) (3). Here, we report on a case of lung adenocarcinoma with brain metastases harboring compound mutations in EGFR exon 21 Leu858Arg and mitogen-activated protein kinase 1 (MEK1) exon 3 lle112Thr and with a high program death ligand 1 (PD-L1) expression who responded well to combination therapy (targeted therapy with furmonertinib, chemotherapy with pemetrexed and lobaplatin, and immunotherapy with tislelizumab), successfully achieving a pathological complete response. We present this case in accordance with the CARE (Case Reporting) checklist.

## Case presentation

A 60-year-old male patient, with a history of smoking, was admitted to our hospital in October 2023 with symptoms of cough and headache[Visual Analogue Scale (VAS) score = 6/10] that persisted for 1 month. The contrast-enhanced computed tomography (CT) scan showed a 4.2-cm × 3.2-cm mass in the left upper lobe with hilar lymph node enlargement. Magnetic resonance imaging (MRI) revealed multiple brain metastases. A left lung biopsy was obtained and established the pathologic diagnosis of pulmonary adenocarcinoma[immunohistochemistry: CK7(+), NapsinA(+)(G), TTF-1(+)(K), CK5/6(−), P40(−), Ki-67(+ approximately 20%), and CD56(−)]. His tumor DNA extracted from the tissue was subjected to DNA sequencing analysis, with genetic testing revealing that the patient had an EGFR exon 21 mutation (c.2573 T>G, p.Leu858Arg 10.5%), MEK1 exon 3 mutation (c.335T>C, p.lle112Thr 40.3%), and a high PD-L1 expression[tumor proportion score (TPS) = 95%]. Based on these data, the patient was diagnosed with stage IVB (cT2bN1M1c1) lung adenocarcinoma.

**Figure 1:**
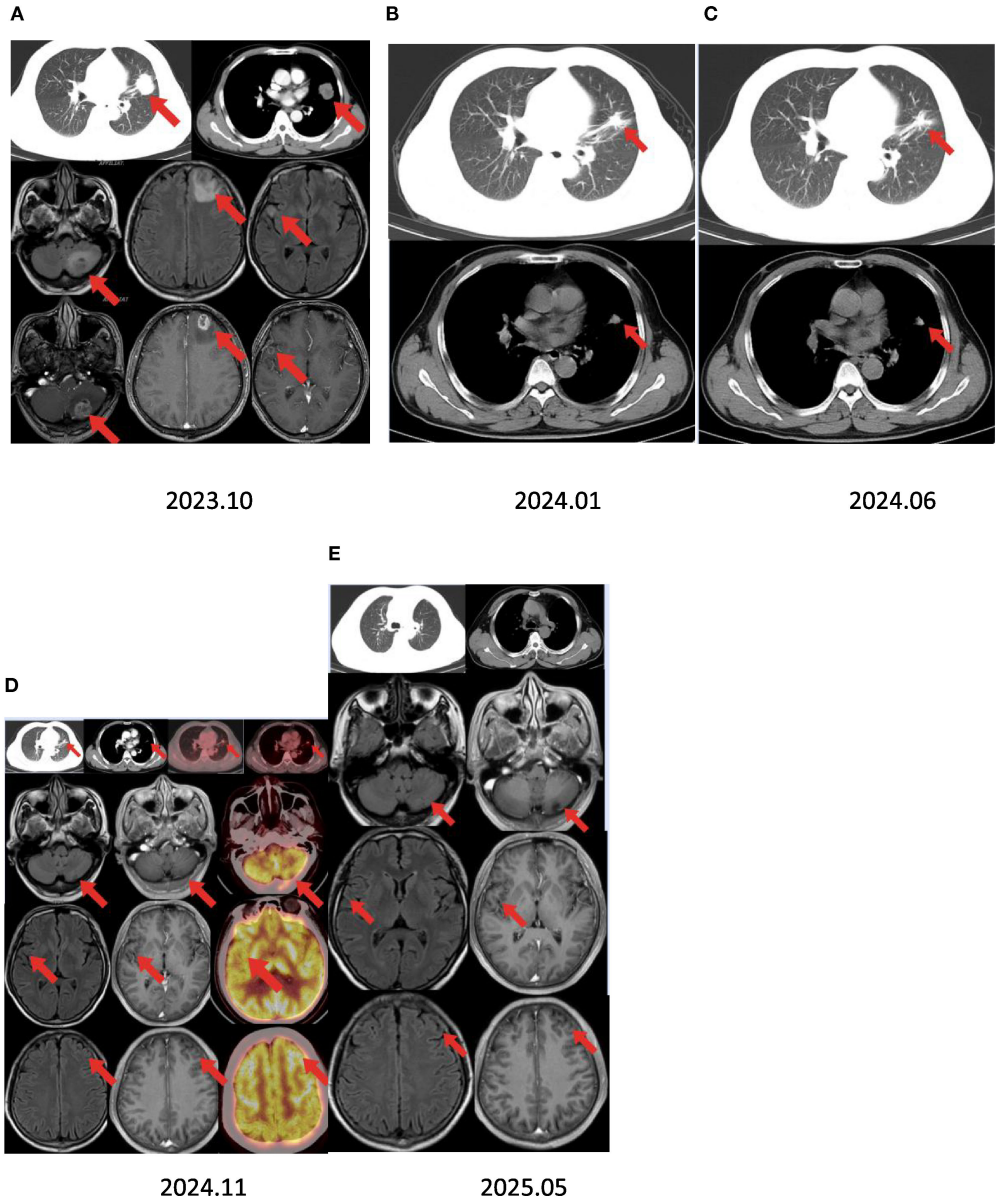
Timeline summary with dynamic imaging of the different therapeutic lines between October 2023 and May 2025. **(A)** Baseline chest CT showing a lung mass in the left upper lobe and brain MRI revealing multiple brain metastases. **(B, C)** Best response under chemotherapy and immunotherapy with a partial response after 2 and 7 months. Assessment showed a partial response of all lung lesions and complete response of the brain lesions after 11 months of furmonertinib. **(D)** Positron emission tomography/CT scan revealing a nodular soft tissue density shadow in the lingual segment of the left upper lobe and increased radioactive concentrations of foci, with a maximum standard uptake value (SUV_max_) of 3.8. There was no increased radioactive concentration of foci in the brain. **(E)** Brain MRI showing no recurrent lesions after 18 months.

**Figure 2:**
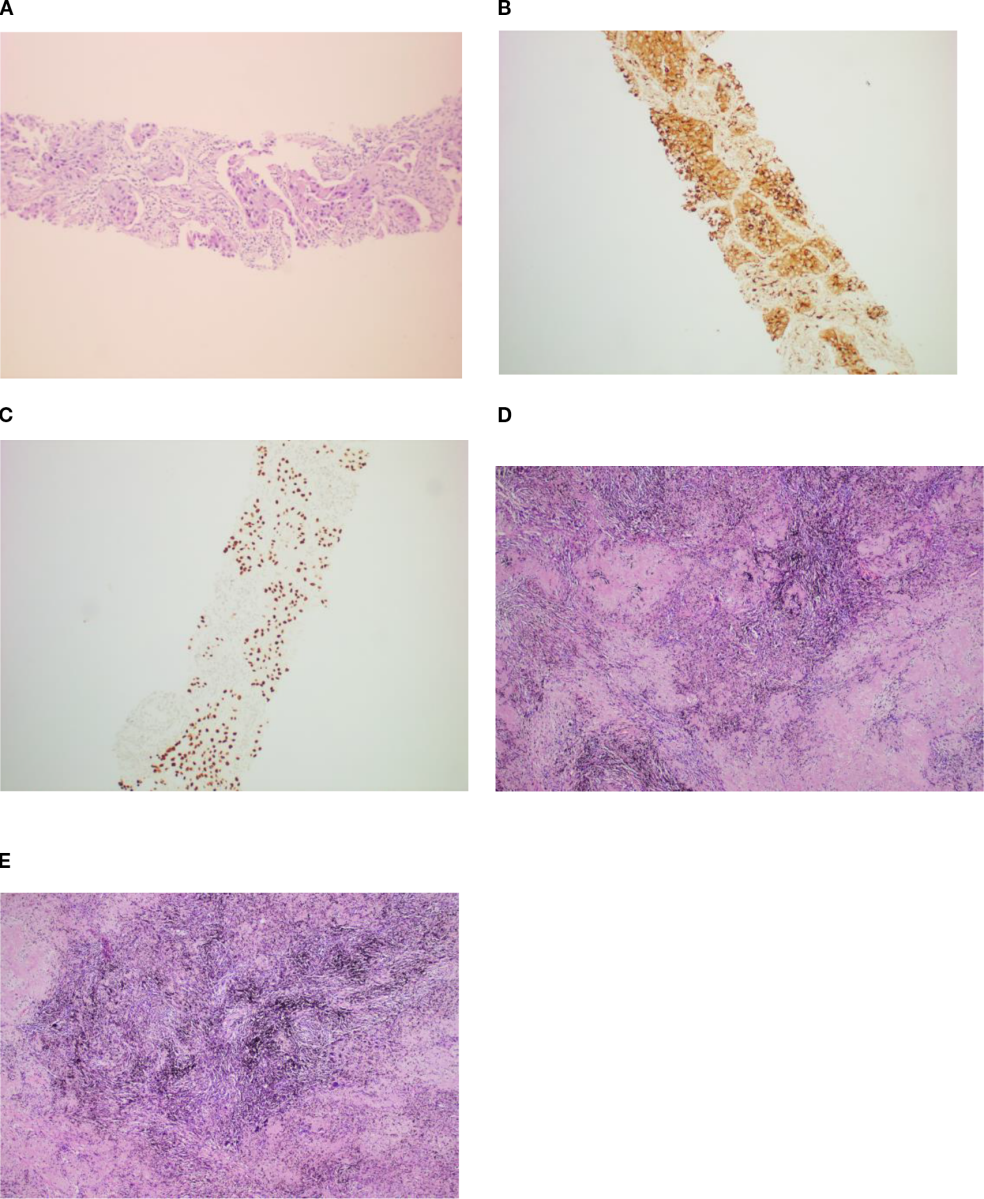
The patient was diagnosed with lung adenocarcinoma via percutaneous CT-guided puncture biopsy **(A)** and immunohistochemistry[CK7(+), NapsinA(+) **(B)**,TTF-1(+) **(C)**, CK5/6(−), P40(−), Ki-67(+ approximately 20%), and CD56(−)]. During postoperative pathology, the residual lung adenocarcinoma was invisible under microscopy, with the area showing coagulative focal necroses, fibrous tissue hyperplasia, and lymphocytic infiltration **(D, E)**. H&E staining, ×10.

The patient started receiving first-line chemotherapy with pemetrexed (800 mg) plus lobaplatin (60 mg) and immunotherapy with tislelizumab after his diagnosis (two cycles of chemotherapy combined with immunotherapy). After a course of treatment, the symptoms of cough and headache resolved (VAS score = 0/10). After 2 months, CT showed a partial response of the lung. He received maintenance targeted therapy with furmonertinib for 11 months. As common treatment-related adverse events (TRAEs), the patient had diarrhea that lasted 1 month and itching for 10 days during this period. No grade ≥3 AEs occurred (**Table 1**). Notably, the patient received no corticosteroids or anticonvulsants during therapy, as he remained neurologically asymptomatic after month 1. The patient’s serial Mini-Mental State Examination (MMSE) scores remained stable at 30/30 throughout treatment. No seizures or focal deficits occurred. In December 2024, the patient underwent a thorough examination at the hospital. Adequate assessment resulted in a complete response of the brain metastases (**Figure 1**). Determining the optimal treatment for the patient is necessary to improve the prognosis; therefore, we conducted a multidisciplinary discussion including oncologists, respiratory physicians, neurosurgeons, radiologists, and radiation oncologists. Finally, the patient underwent thoracoscopic left upper lobectomy and lymph node dissection for lung cancer, and pathological examination confirmed ypT0N0M0: 0/13 lymph nodes involved (stations 5–7 and 10–12 dissected) (**Figure 2**). Minimal pleural adhesion (grade 1, Goldberg classification) and minor bleeding were observed during the procedure. No air leaks occurred after the procedure, and no signs of acute respiratory distress syndrome (ARDS) were present.

**Table 1:** Adverse events according to the Common Terminology Criteria for Adverse Events (CTCAE) v5.0 criteria.

Adverse event	Max grade	Evidence	Management	Outcome
Diarrhea	2	4–6 stools per day	Taking Imodium	Resolved in 4 weeks
Hypothyroidism	–	Normal index	–	–
Pneumonitis	–	Serial CT: no GGO/fibrosis	–	–
Hepatic insufficiency	1	ALT = 69 U/L AST = 53 U/L	Taking liver protective drugs	Resolved in 3 days
Neutropenia	–	Normal index	–	–
Pruritus	2	Widespread and intermittent	Taking loratadine	Resolved in 10 days
Myocardial injury	–	Normal index	–	–
Creatinine increased	1	>ULN-1.5 × ULN	Taking kidney-conserving drugs	Resolved in 7 days

*GGO*, ground-glass opacity; *ALT*, alanine transaminase; *AST*, aspartate transaminase; *ULN*, upper limit of normal.

## Discussion

The central nervous system (CNS) is a common site for metastasis originating from lung cancer, making it the most prevalent type of brain metastasis (4). In NSCLC, approximately 10%–30% of patients are found to have brain metastases at the time of their initial diagnosis, a figure that tends to increase as treatment progresses (5). The presence of brain metastasis is frequently associated with a poor prognosis, often leading to a fatal outcome for patients with lung cancer (6). Symptoms of brain tumors can be categorized into general and localized manifestations, and individuals frequently exhibit both types of symptoms. Common generalized symptoms may include headaches, cognitive dysfunction, renal girdle changes, and gait disorders. Unilateral and localized manifestations include hemiparesis, speech impairment, and visual field defects. Brain metastases can be treated with local therapy, such as surgery, radiotherapy, or systemic therapy with anticancer drugs. The selection of the therapeutic approach is influenced by the histological classification, the patient’s overall health, and the quantity and dimension of the brain metastases (7).

The American Society of Clinical Oncology (ASCO)–Society for Neuro-Oncology (SNO)–American Society for Radiation Oncology (ASTRO) guidelines (8) indicate that, for individuals with brain metastases, surgical intervention can be a viable choice when various factors are taken into account. However, patients presenting with multiple brain lesions and poorly managed systemic diseases might see limited advantages from surgery unless the systemic condition is effectively addressed. For single brain metastasis, the European Association of Neuro-Oncology (EANO)–European Society for Medical Oncology (ESMO) guidelines (9) emphasize the preference for neurosurgical resection over any other intervention in patients with controlled systemic disease. For patients with symptomatic brain metastases, consideration should be given to the use of localized treatments (such as radiosurgery or a combination of approaches). It is advisable not to postpone local interventions, even in patients with asymptomatic brain metastases. Nonetheless, it is justifiable to postpone local treatment for individuals with asymptomatic brain metastases who are undergoing therapy with EGFR TKIs, e.g., *ALK* TKIs, including pemetrexed combined with platinum and pembrolizumab for those who test positive for PD-L1. It is crucial that the choice to postpone local treatment is made following a comprehensive evaluation by a team of specialists, weighing the advantages and disadvantages that the patients might face (7).

Chemotherapy and targeted therapies have limited effectiveness against brain tumors due to the blood–brain barrier (BBB) (10). However, recent advancements in the availability of drugs that target brain metastases have made it sensible to consider systemic therapy prior to local interventions for cases of asymptomatic brain metastases.

The FLAURA study demonstrated that osimertinib, a third-generation EGFR TKI, is beneficial for patients presenting with brain metastases, indicating an effective rate of intracranial disease control (11). Moreover, it has been demonstrated that furmonertinib, as an initial therapy, outperforms gefitinib in terms of CNS PFS, CNS objective response rate, and the extent of CNS response in individuals with EGFR-mutated NSCLC and CNS metastases (1).

Immune checkpoint inhibitors play a crucial role in the treatment of lung cancer that lacks driver mutations, while the presence of PD-L1 on tumor cells serves as an indicator of the effectiveness of anti-PD-1/PD-L1 treatments. Clinical trials within the KEYNOTE framework (12) (specifically KEYNOTE-021, KEYNOTE-189, and KEYNOTE-407) indicated that the combination of pembrolizumab and chemotherapy resulted in improved survival rates compared with chemotherapy alone, irrespective of whether a patient had brain metastases at the start of treatment. The effectiveness of immune checkpoint inhibitors as an initial therapy for advanced or metastatic non-squamous NSCLC in individuals with brain metastases was further supported by the findings from CheckMate 817 (13) and CheckMate 227 (14). In the context of locally advanced or metastatic non-squamous NSCLC, the combination of Tislelizumab with platinum-based chemotherapy and pemetrexed demonstrated a favorable PFS profile and was generally well accepted by patients, exhibiting manageable levels of toxicity.

Currently, the CTONG0803 study and the BRAIN study have confirmed that icotinib is superior to radiotherapy in the control of intracranial lesions in NSCLC patients with EGFR-positive multiple brain metastases (15, 16). Determining the optimal treatment for the patient is necessary to improve the prognosis; therefore, we conducted a multidisciplinary discussion including oncologists, respiratory physicians, neurosurgeons, radiologists, and radiation oncologists. Considering the multiple metastases in the brain, the neurosurgeons did not recommend surgery, while a radiation oncologist advised the patient to undergo brain radiotherapy. After understanding the final treatment regimen, the patient refused brain radiotherapy. He received chemotherapy, immunotherapy, and targeted therapy. After a course of treatment, the symptoms of cough and headache resolved, and adequate assessment resulted in a complete response of the brain metastases after 13 months. The safety of the triple-combination therapy remains a concern. While the FURLONG trial reported 28.4% grade ≥3 TRAEs with furmonertinib monotherapy (17), our regimen added only grade 1–2 toxicities. Finally, the patient underwent thoracoscopic left upper lobectomy and lymph node dissection for lung cancer, and pathological examination confirmed ypT0N0M0. No tumor recurrence was found in the brain and the lung during follow-up.

## Conclusion

For brain metastases from lung cancer, both surgery and radiotherapy are the basic treatment options. However, it is necessary to assess the patient’s general condition and to determine who qualifies for treatment as it may not be optimal in certain cases. With drug development and the updated data on drug therapy, chemotherapy, molecular targeted therapy, and immunotherapy are currently available as effective treatment methods. Multidisciplinary treatment has become increasingly important for application of the latest information in clinical practice.

## Data Availability

The datasets presented in this study can be found in online repositories. The names of the repository/repositories and accession number(s) can be found in the article/supplementary material.
